# Severity markers in acute pancreatitis

**DOI:** 10.1186/cc14469

**Published:** 2015-03-16

**Authors:** S Jalkanen

**Affiliations:** 1 University of Turku, Finland

## Introduction

CD73/ecto-5'-nucleotidase is an enzyme that generates adenosine, which dampens inflammation and improves vascular barrier function in several disease models. CD73 also circulates in a soluble form in the blood [1]. We studied whether levels of soluble form of CD73 and cytokines/chemokines predict the development of organ failure in acute pancreatitis [2,3].

## Methods

Altogether, 161 patients with acute pancreatitis (107 were subclassified according to the revised Atlanta criteria into mild, 29 into moderately severe and 25 into severe forms) were studied. Serum and blood cell samples were collected at admission. Protein levels of soluble form of CD73 in serum were determined using a novel enzyme-linked immunosorbent assay, activity of soluble form of CD73 using radioactive enzyme assays, and CD73 messenger RNA levels from leukocytes using quantitative PCR. Serum levels of 48 cytokines and growth factors were determined using Bio-Plex Pro Human Cytokine Assay 21-plex and 27-plex magnetic bead suspension panels.

## Results

Activity and protein concentration of soluble form of CD73 and messenger RNA level of CD73 all decreased along with the disease severity (*P ≤*0.01 for all). The activity of soluble form of CD73 at admission predicted the development of severe pancreatitis in different groups of the patients. Especially, activity of soluble form of CD73 was better than C-reactive protein or creatinine in predicting the severity of pancreatitis in the group of patients without any signs of organ failure at admission. In subgroup analyses of patients with severe pancreatitis and without organ dysfunction upon admission, IL8, hepatocyte growth factor and granulocyte colony-stimulating factor (G-CSF) levels predicted the development of severe pancreatitis, with G-CSF being the most accurate cytokine.

## Conclusion

Activity of soluble form of CD73 and levels of certain cytokines at admission to the hospital have prognostic value in predicting the development of the severe form of acute pancreatitis. The possibility that combining them with other prognostic markers might improve prognostic accuracy requires further studies.
